# Features of Gene Regulation in Violation of the Inflammatory Response of Monocyte-like Cells Bearing Mitochondrial Mutations Associated with Atherosclerosis

**DOI:** 10.2174/0109298673303008240829075444

**Published:** 2024-09-12

**Authors:** Alexander N. Orekhov, Nikita G. Nikiforov, Alexander D. Zhuravlev, Svetlana S. Verkhova, Andrey V. Omelchenko, Daria D. Borodko, Vasily N. Sukhorukov, Vasily V. Sinyov, Igor A. Sobenin

**Affiliations:** 1 Institute of General Pathology and Pathophysiology, 8 Baltiiskaya Street, 125315, Moscow, Russia;; 2 Petrovsky Russian National Center of Surgery, 2 Abrikosovsky Lane, 119991, Moscow, Russia;; 3 National Medical Research Center of Cardiology, 15a Academician Chazov Street, 121552, Moscow, Russia

**Keywords:** Cybrid, cytokine, inflammatory stimulation, intolerant response, master regulator, mitochondrial mutations, signaling pathway, trained immunity

## Abstract

**Аims:**

This research aimed to study the features of gene regulation of the inflammatory response in cells carrying mitochondrial mutations associated with atherosclerosis.

**Background:**

Inflammation plays an important, if not decisive, role in the occurrence of atherosclerotic lesions and then accompanies it throughout its further development. Thus, atherogenesis is a chronic inflammatory process. Chronification of inflammation is a consequence of disruption of the normal inflammatory response at the cell level of the vascular wall.

**Objectives:**

In this study, we used cytoplasmic hybrids or cybrids carrying atherosclerosis-associated mitochondrial mutations to study gene regulation of inflammatory response. The main goal of the study was to identify the key genes responsible for the impaired inflammatory response revealed for some cybrids.

**Methods:**

Inflammatory stimulation of cybrids was induced with bacterial lipopolysaccharide, and assessed through secretion of pro-inflammatory cytokines CCL2, IL8, IL6, IL1b. A transcriptome analysis was performed to identify the key genes (master regulators) in the normal (tolerant) and intolerant response of cybrid cells.

**Results:**

Normal inflammatory response after re-stimulation elicited a much smaller secretion of pro-inflammatory cytokines. In an intolerant response, the level of secretion upon re-stimulation was the same or even higher than after the first stimulation. Normal and intolerant responses differed significantly both in terms of the number of signaling pathways involved and qualitatively, since the signaling pathways for normal and intolerant responses are completely different. Master regulators controlling normal and intolerant inflammatory response were identified. For a normal response to the first inflammatory stimulation, no common master up-regulators and 3 master down-regulators were identified. The reverse situation was observed with the intolerant inflammatory response: 6 master up-regulators, and no master down regulators were identified. After the second inflammatory stimulation, no master regulator common to all studied cytokines was found. Thus, key genes involved in the development of intolerant inflammatory response have been identified. In addition, other key genes were identified that were initially associated with an intolerant inflammatory response and thus determine disorders of the inflammatory reaction leading to chronification of inflammation.

**Conclusion:**

We identified disturbances in gene associated with the development of intolerant immune response that may be relevant to atherosclerosis. Key genes responsible for the chronification of inflammation were discovered.

## 1 INTRODUCTION

Inflammation is a natural response of the innate immune system that evolved primarily to defend the host organism against pathogens [1-6]. There are cellular mechanisms involved in acute inflammation, which are very important in protecting the body from infection and tissue damage [7-12]. However, prolonged or excessive inflammation can lead to tissue damage and chronic inflammatory conditions [13-17]. Immune tolerance is an important regulatory feature of the normal immune response [18-22]. We have shown that the response of human monocytes and macrophages to inflammatory stimulation is impaired in atherosclerosis [23-28]. Normal inflammation must resolve quickly due to existing effective mechanisms, including tolerance resulting from repeated inflammatory stimulation [29-31]. This process ensures that the secretion of pro-inflammatory molecules, such as cytokines, by macrophages, is significantly lower upon repeated inflammatory stimulation compared to the first stimulation [31, 32]. If the inflammatory response is impaired, tolerance is reduced or absent, and cytokine secretion upon repeated stimulation does not decrease but increase significantly [33, 34]. This can lead to the chronification of inflammation.

Atherosclerosis is an inflammatory disease based on local chronic inflammation of the arterial wall [35, 36]. There can be many factors that trigger an inflammatory reaction in a vessel [37, 38]. These are mainly pathogen-associated molecular patterns (PAMPs) [39] and damage-associated molecular patterns (DAMPs) [40]. PAMPs are relatively non-specific, highly conserved, pathogenic molecular structures expressed in pathogens, and their products [41]. DAMPs are a large number of related intracellular proteins or nucleic acids [42]. In the case of atherosclerosis, DAMPs include modified LDL circulating in the blood of patients and locally accumulating in the vascular wall [43, 44].

The reasons for the chronification of the inflammatory response in atherosclerosis are not clear [45, 46]. Based on our own data, we hypothesize that mitochondrial dysfunction may play a key role in this pathological reaction, and one of the reasons for such dysfunction may be mitochondrial mutations [47-51].

Previously, we have identified human mitochondrial DNA mutations (genome variants) associated with atherosclerosis [52, 53]. To study the role of these mutations in atherogenesis, cytoplasmic hybrids or cybrids, carrying atherosclerosis-associated mitochondrial mutations were created from cultured monocyte- like THP-1 cell line. To create a cybrid line, the cell line’s own mtDNA was suppressed, followed by the fusion of the THP-1-derived ρ0 cells with platelets containing mtDNA from different patients with atherosclerosis [54].

We recently reported that some atherosclerosis-associated mutations may be responsible for the intolerant immune response of cybrids [55]. In the present work, we performed a transcriptome analysis to study the signaling pathways in the normal (tolerant) and abnormal (intolerant) response of cytokine secretion.

## 2 MATERIALS AND METHODS

### 2.1 Cybrid Lines

Cybrid lines were obtained using the technique of King and Attardi [56], adopted in application to the THP-1 cell line [54]. The THP-1 cell line was purchased from the American Type Culture Collection (ATCC, USA). The principle of the standard method is based on the use of inhibitors of mtDNA replication by ethidium bromide to obtain mitochondrial-free (ρ0) cells from the original THP-1 cell line. Cells were cultured in RPMI-1640 medium (GIBCO) containing 300 mg/ml L-glutamine 2x10^-5^ M 2-mercaptoethanol, 110 μg/ml sodium pyruvate, 2500 mg/l glucose, 50 U/ml penicillin-streptomycin, and 10% fetal bovine serum. The cells were maintained in culture for 18 weeks, with passage every 2-3 days. Next, ethidium bromide was excluded from the growth medium, and the resulting mitochondrial cells were cultivated in the growth medium supplemented with 200 μg/mL uridine. To obtain cybrid cultures, the PEG-fusion technique was used, which ensures the penetration of mitochondria into ρ0 cells by forming pores in the cellular cytoplasmic membranes. The use of platelets greatly simplifies the protocol for obtaining cybrid lines, since platelets contain only the mitochondrial genome. Platelets were isolated from the whole blood of donors by centrifugation on a Ficoll-Paque (GE Healthcare, IL, USA) density gradient. Sodium citrate in physiological saline was added to the blood obtained from donor patients in a 1:1 ratio. The resulting mixture was centrifuged for 20 min at 200 g at 12°C. Three quarters of the supernatant (plasma) was taken and centrifuged for 20 min at 1500 g at a 15°C, the supernatant was removed, and 11 ml of physiological saline was added to the remaining sediment. The resulting platelet fraction was stored for cryopreservation after addition of 15 ml of sterile DMSO and 3 ml of fetal calf serum. Next, cells were subjected to controlled freezing in cryovials (10°C/min) for 8 hours until a temperature of -80°C was reached. The samples were then stored in liquid nitrogen. In this study, we used 8 lines of cybrids and the maternal line THP-1.

### 2.2 Pro-Inflammatory Response

In order to compare the pro-inflammatory response of cybrids, inflammatory stimulation with bacterial lipopolysaccharide (LPS) was used. LPS (1μg/ml) was added to the cybrid culture for 4 hours (1^st^ stimulation, or “hit”). Cells were then washed with PBS, and LPS was added for another 20 hours (2^nd^ hit). Upon completion of the experiment, the secretion of cytokines CCL2, IL8, IL6, and IL1b was measured using ELISA.

The basal secretion before the first stimulation (1^st^ hit) was considered to be the secretion of the cytokine by cells without LPS. The basal secretion prior to the second stimulation (2^nd^ hit) was considered to be the secretion from the cells that had received the first stimulation (1^st^ hit). The significance of differences in the secretion of cytokines was assessed by a paired t-test after applying the appropriate bootstrap procedure.

### 2.3 Sequencing

For transcriptome analysis, total RNA was extracted from the provided samples using the manufacturer's TRIzol protocol (Invitrogen). The samples were treated with a recombinant duplex-specific nuclease (Eurogene, Russia) to remove genomic DNA contamination. The quality of the total RNA from 20 samples was assessed by horizontal gel electrophoresis.

Using the TruSeq mRNA Stranded reagent kit (Illumina, USA), poly(A+) fraction enrichment was performed on total RNA from samples, followed by cDNA synthesis with random primers. The resulting cDNA was used to prepare libraries compatible with Illumina sequencing technology.

The quality of the obtained libraries was checked using a Fragment Analyzer instrument. Quantitative analysis was performed by qPCR. After quality control and DNA quantity assessment, the library pool was sequenced on an Illumina NovaSeq 6000 instrument (read length of 150 bp from both ends of fragments). FASTQ files were obtained using bcl2fastq v2.20 Conversion Software (Illumina, USA). The quality score format was Phred 33.

The quality control of sequencing results performed using FastQC [57] showed high read quality and the presence of a small amount of adapter sequences. Prior to downstream analysis, read filtering based on length and quality, as well as adapter trimming, was performed using TrimGalore! and Cutadapt [58].

### 2.4 Transcriptome Analysis

To assess the quality of the obtained reads, FastQC v0.11.9 software was employed. The quality scores of the reads were encoded using the Phred33 scale. The evaluation of the quality indicated that the reads had sufficient quality for subsequent analysis within the STAR protocol without requiring any trimming.

For read alignment and mapping, the STAR aligner was employed. The reads were mapped to the human GRCh38 assembly. To further evaluate the quality of the aligned reads, RSeQC was utilized. This additional assessment ensured the reliability and accuracy of the aligned reads. To obtain gene expression counts, the STAR protocol used for mapping included two specific options: “—quantMode GeneCounts” and “--sjdbGTFfile GRCh38 annotation”. This step generated count files, which were then used to construct a count matrix for DGE analysis with DESeq2 package for R. Pathway enrichment analysis was performed using the R package enrichR. The pipeline of the search for master regulators of signaling pathways was carried out on the geneXplain platform [59] based on the compilation of interaction networks in accordance with the GeneWays database [60] and the search for genes located at a distance of no more than 10 higher levels from these genes, with parameters Score > 0.2, FDR < 0.05 and Z-score > 1.0. All statistical tests were carried out with adjusted *p*-value < 0.05.

Further analysis was carried out using original programs written in the R programming language using the DESeq2 package (v1.42.0) and the tidyverse family of packages (v2.0.0). The count table was normalized to the size of the genomic library using the counts() function from the DESeq2 package, for which the “median of ratios” method is used in accordance with the recommendations of the package developers.

Next, descriptive statistics were calculated for each sample, and it was determined that the 3^rd^ quartile of Q3 normalized counts lies in the region from 1100 to 1800 transcripts. Thus, it was accepted that a gene is considered to be expressed at the proper level if the number of normalized counts for it on average exceeds 1000 transcripts. The samples were divided into groups according to the type of immune response (tolerant or intolerant) for each cytokine for which this type of immune response was manifested (IL1b, IL6, IL8, CCL2).

Next, by filtering genes, a set of genes with an intolerant response for each cytokine was formed separately, with the selection of all genes from the control samples (without any influence) whose expression was above 1000 transcripts. Then, a set of genes with a tolerant response for each cytokine was generated separately, with the selection of all genes from the control samples (without any influence) whose expression was above 1000 transcripts. Next, a comparative analysis of the set of genes with an intolerant response and the set of genes with a tolerant response was carried out. Onlyy those genes that are in the set with an intolerant responsewere selected, butthose thath are not in the set with a tolerant respons were selectede. Then, the sets of genes associated with the intolerant response for these cytokines obtained for 4 cytokines were intersected to obtain a set of genes that are expressed only during the intolerant response. In this way, 66 unique candidate genes were obtained that are associated with intolerant immune responses. These genes were ordered in descending order of expression level, and from this ordered set of genes, the TOP-10 genes with the highest expression were selected.

Functional annotation of the named genes was obtained from the Entrez database using the RDAVIDWebService (v1.0.0) software package.

### 2.5 Statistical Analysis

Biological copies in the present study ranged from 3 to 9 replicates depending on the cytokine (IL1b, IL6, IL8, CCL2) for each experiment. The resampling and randomization methods used in the study are best practices in assessing statistical patterns in modern scientific research. In particular, bootstrap analysis was used, which contributes to a better statistical assessment of the significance of differences in central tendencies such as mean, median, and mode. The resulting differences had adjusted *p*-values significantly lower than the critical level of 5% and ranged from 2×10^-5^ to 0.03, which indicates a significant statistical difference.

## 3 RESULTS

### 3.1 Intolerant Response of Cytokine Secretion to Inflammatory Stimulation

The atherosclerosis-associated mitochondrial DNA (mtDNA) mutations (genome variants) identified in human atherosclerotic lesions and blood cells have been described previously [52, 53, 61]. To study the role of these mutations in atherogenesis, we created several cybrid cell lines based on monocyte-like THP-1 cells. The obtained cybrid cells differ by the profile of atherosclerosis-associated mitochondrial mutations [54]. Eight cybrid lines were obtained in this way, and paternal THP-1 lines were used in the present work.

To compare the pro-inflammatory response of cybrids, the secretion of 4 cytokines (CCL2, IL8, IL6, and IL1b) was measured. These four pro-inflammatory cytokines were chosen for study because they have different roles in the inflammatory response and differ in their mechanism of response to inflammatory stimuli. In addition to the activation of cytokine secretion in response to inflammatory stimulation, we assessed the tolerance of monocyte-like cells upon repeated LPS stimulation. Typically, re-stimulation elicits a much smaller response in the form of the secretion of pro-inflammatory cytokines, while an equal or greater response indicates intolerance. Fig. (**1**) demonstrates the typical normal (tolerant) and intolerant pro-inflammatory response of secretion of the studied cytokines.

### 3.2 Transcriptome Analysis

#### 3.2.1 Gene Expression

**Fig. (1):**
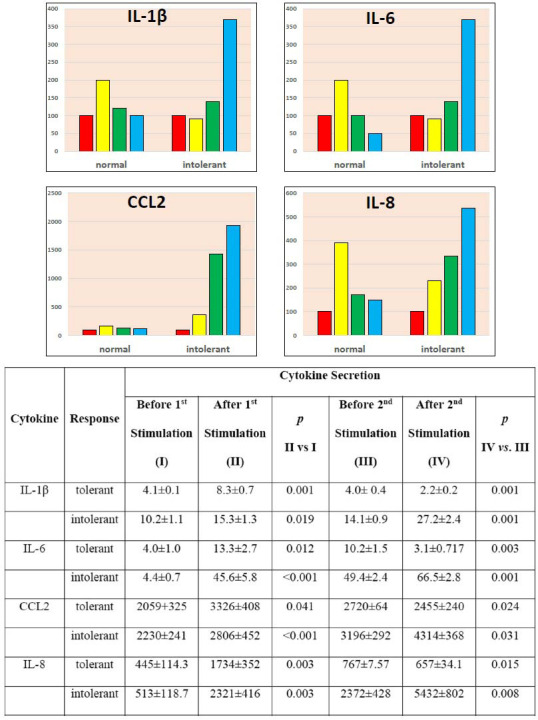
Normal (tolerant) and intolerant response of cytokine secretion to inflammatory stimulation. **Note:** The results of a typical experiment are presented. In total, more than 10 similar experiments were carried out. The mean values and standard deviations of independent experiments are given. The significance of the differences were assessed by using a t-test after applying the appropriate bootstrap procedure. The vertical axis shows the relative secretion of the cytokine as a percentage of the basal secretion (before stimulation) taken as 100% (red bars). Yellow bars are cytokine secretion after the first stimulation. Green bars are cytokine secretion before the second stimulation. Blue bars are cytokine secretion after the second stimulation.

**Fig. (2):**
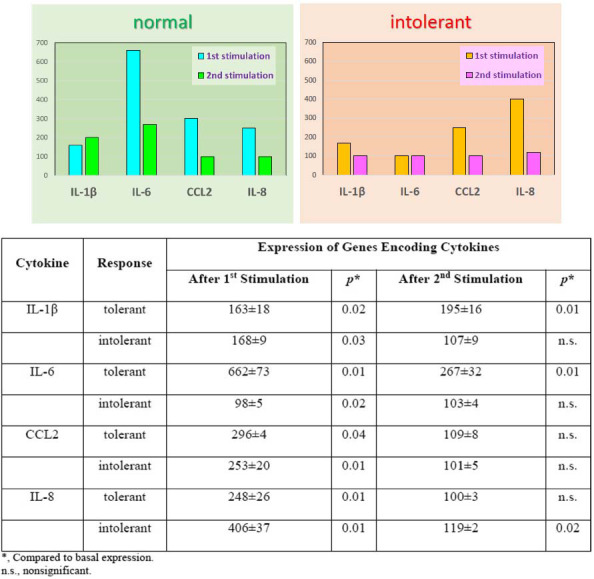
Relative expression of genes encoding cytokines in normal and intolerant cell response. **Note:** The mean values and standard deviations of independent experiments are given. A paired t-test assessed the significance of differences after applying the appropriate bootstrap procedure. The number of biological replicates for each cytokine in a normal response was at least 3. For an intolerant response, the number of biological replicates was as follows: IL-1β - 3, IL-6 - 4, CCL2 - 9, IL-8 - 8. The actual adjusted *p*-value levels ranged from 2×10-5 to 0.03 for the differences found. The vertical axis shows the relative expression of the cytokine as a percentage of the basal expression (before stimulation) taken as 100%.

Transcriptome analysis was used to study the mechanisms of differences between normal and intolerant responses. We first evaluated the expression of genes encoding the corresponding cytokines. Expression after the inflammatory stimulation was presented as a percentage of expression before stimulation (taken as 100%). Gene expression data for normal and intolerant responses are shown in Fig. (**2**). We found a considerable discrepancy between the expression and the secretion of cytokines in the intolerant response. With a tolerant response, cytokine secretion was significantly lower, and with an intolerant response, it was significantly higher after the second stimulation compared to the first (Fig. **1**). At the same time, the expression of the genes encoding these cytokines changed in the most bizarre way (Fig. **2**).

- After the first stimulation, IL1b expression increased 1.6-fold in cells with a normal response and 1.7-fold with an intolerant response. After the second stimulation, IL1b expression increased 2-fold in cells with a normal response and did not change in cells with an intolerant response.

- After the second stimulation, the expression of IL 6 increased 2.7 times in cells with a normal response and did not change in cells with an intolerant response.

- After the first stimulation, CCL2 expression increased 3-fold in cells with a normal response and 2.5- fold with an intolerant response. After the second stimulation, the expression of CCL2 in cells with a normal response and with an intolerant response does not change.

- Expression of the IL 8 gene increased 2.5 times after the second stimulation in cells with a normal response and did not change in cells with an intolerant response. After the first stimulation, IL8 expression increased 4-fold in cells with a normal response and 1.19-fold in cells with an intolerant response.

Thus, assessment of the expression of genes encoding the cytokines under study cannot explain the change in their secretion since no clear relationship has been found between gene expression and cytokine secretion. The secretion of cytokines depends not only on the regulation of the genes encoding these cytokines, so we examined the signaling pathways and identified key genes (master regulators) involved in the inflammatory response.

#### 3.2.2 Signaling Pathways

For each of the four studied cytokines, transcriptomes were selected from cybrids with a normal and intolerant response. Transcriptomes were compared before and after stimulation, and up-and down-regulated signaling pathways were identified. The number of signaling pathways identified is presented in Table **1**. The inflammatory stimulation triggered a different number of up-and down-regulated pathways for different cytokines. The number of signaling pathways was also different for normal and intolerant responses of the same cytokine.

Signaling pathway characteristics of all cytokines specific only for normal or only for intolerant responses were identified. Many up-regulated signaling pathways have been identified; however, downregulated pathways that are common (coinciding) for all cytokines have not been identified with any stimulation. The qualitative difference between normal and intolerant responses is presented in Supplementary Table **1**. In cybrids with a normal response, the first inflammatory stimulation triggered only 3 unique (characteristic only for a normal response) signaling pathways. After the second stimulation, 13 pathways weretriggered. The opposite was observed in cells with an intolerant response: after the first stimulation, 50 signaling pathways were activated, and after the second stimulation, only 5.

Thus, transcriptome analysis revealed that normal and intolerant responses differed significantly both quantitatively, in terms of the number of signaling pathways involved, and qualitatively, since the signaling pathways for normal and intolerant responses are completely different.

#### 3.2.3 Master Regulators Involved in the Intolerant Response

Next, we identified the master regulators of normal and intolerant inflammatory responses. From a large set of known master regulators, we selected those that matched all four studied cytokines. In the case of a normal response to the first inflammatory stimulation, no common master up-regulators were found, while 3 master down-regulators were identified (Table **2**).

After the second inflammatory stimulation, no master regulator common to all studied cytokines was found.

Thus, we found the key genes involved in the intolerant inflammatory response.

The reverse situation was observed with an intolerant inflammatory response. Six masters up regulators were found (Table **3**), but no master-down regulators were identified.

#### 3.2.4 Key Genes Responsible for Intolerant Response

At the final stage, we discovered genes in unstimulated cells that may predispose to an intolerant response. For this purpose, we compared cultures of cybrids that demonstrated an intolerant response with cybrids with a normal response. In initially unstimulated cells, highly expressed genes were identified that are characteristic only of those cultures that, upon further inflammatory stimulation, demonstrated an intolerant cytokine response. Of the 66 genes identified in this way, the top 10 were selected and are shown in Supplementary Table **2**.

The identified genes encode a variety of proteins involved in different cellular functions. At first glance, these proteins do not have anything in common; therefore, special studies will be required to clarify their participation in the disruption of the normal inflammatory response. We have started this work, which will consist mainly of genome editing. It will take time to complete the research and analyze the results. The obtained data will be published in our subsequent articles.

## 4 DISCUSSION

In a properly regulated inflammatory response, the immune system detects the presence of a threat, triggers an inflammatory response, eliminates the threat, and then resolves the inflammation [62, 63]. However, in certain cases, the inflammatory response becomes imbalanced, leading to the chronification of inflammation or an exaggerated immune response, which can result in various disorders [64, 65]. Atherosclerosis is among the well-known disorders associated with abnormalities in the inflammatory response [66]. Chronic inflammatory diseases are characterized by persistent inflammation lasting for an extended period of time. In these conditions, the immune system mistakenly triggers an inflammatory response, even in the absence of an apparent threat or after the initial cause of inflammation has been resolved. Dysregulated cytokine production is one of the main key points about disorders of the inflammatory response in chronic inflammatory diseases [67]. We do not consider such extreme types of dysregulated inflammatory responses as cytokine deficiency or cytokine storm. We are interested in the mechanisms of inflammation chronification when the immune system can become dysregulated, resulting in the sustained secretion of cytokines. In particular, this occurs with intolerant secretion of cytokines. The term “intolerant cytokine secretion” itself is not widely used in scientific literature or medical practice. However, the concept of dysregulated cytokine release is well-recognized and studied in the context of various immune-mediated disorders.

The term “trained immunity” has been used in the literature since 2011 [68], which is also characterized by the increased production of pro-inflammatory cytokines upon a secondary inflammatory stimulation [29, 69-72]. Trained immunity or “innate (learned) memory” is considered a more effective innate memory response; that is, this phenomenon can be considered a useful tool [69, 73]. We do not use the term “trained immunity” but refer to the increase in secretion of pro-inflammatory cytokines upon a secondary inflammatory stimulation as an intolerant inflammatory response because we believe that we are dealing with a violation of the inflammatory response leading to chronification of inflammation. Of course, this is not useful. We have shown that not all cybrids obtained from various atherosclerotic patients have an intolerant response. There were also cybrids with a normal response, which indicates a different predisposition of different cell lines to the chronification of inflammation. According to our data, the intolerant response is associated with certain mutations in the mitochondrial DNA [55, 56].

Trained immunity and an intolerant immune response manifest themselves in the same way, namely as increased secretion of pro-inflammatory cytokines upon repeated inflammatory stimulation. Nevertheless, we believe that the term “trained immunity” is more suitable for infectious pathologies where this phenomenon plays a positive role, and “intolerant immune response” should be used for non-infectious diseases in the occurrence and development of which an intolerant response plays a clearly negative role causing chronification of inflammation.

The mechanisms of trained immunity have been successfully studied over the past decade. Pathogens that induce trained immunity are recognized by the cell through various pattern-recognition receptors [74]. Endogenous stimuli that induce trained immunity can activate various receptors [75, 76]. Binding to these receptors activates metabolic processes, causing epigenetic modifications [77]. The increased activity of trained cells is due to the increased availability of the corresponding genes for transcription after restimulation [77, 78]. Increased accessibility is the result of epigenetic modifications that unfold chromatin and expose immune-related genes.

How does the negative role of trained immunity manifest itself in non-infectious diseases and, in particular, in atherosclerosis? Ideas about trained immunity and its mechanisms are the result of studies on human monocytes as well as studies on mice. However, no data were obtained on monocyte-macrophages of patients that would indicate that trained immunity contributes to the development of atherosclerotic diseases [33]. Our own data support these findings, not only in relation to atherosclerosis but also in other diseases based on chronic inflammation. According to our unpublished data, in most cases, the phenomenon of trained immunity could not be detected in the primary culture of monocyte macrophages isolated from the blood of healthy individuals and patients with various inflammatory diseases.

In this work, we observed an intolerant immune response (trained immunity) in cybrids. At least half of the obtained cybrids showed an intolerant response, while other cybrids responded to inflammatory stimulation in a normal manner (decreased cytokine secretion on repeated stimulation). Naturally, we were intrigued by the question of why we and others did not observe an intolerant response in the primary monocyte-macrophages isolated from the blood of patients, although, as we know, these cells also carry atherosclerosis-associated mitochondrial mutations. There is no direct answer to this question, but some speculation can be offered. Apparently, in the absence of infection, an intolerant response is detrimental to the body because it provokes chronic inflammation. As a defense against this, differentiating and mature immune cells may undergo epigenetic regulation, leading to the elimination of the conditions for an intolerant response. Cybrids never existed in the body, so they have no epigenetic protection, and nothing prevents mitochondrial mutations from contributing to an intolerant response.

Another possible and most likely explanation for the absence of an intolerant response in the population of monocyte-macrophages isolated from the blood of patients may be that the proportion of cells with an intolerant response is low, and in the general population, their intolerant response cannot manifest; the intolerant response may go unnoticed against the background of the prevalence of cells with a normal response. However, “intolerant” cells entering the arterial system can cause local chronic inflammation, which is what is observed in atherosclerosis.

In the present work, we have revealed a dramatic difference in gene regulation in normal and intolerant responses to inflammatory stimulation. Many signaling pathways have been identified that do not overlap with normal and intolerant responses. Moreover, key genes (master regulators) were found that are characteristic only for a normal and only intolerant response. The fact that in the case of a normal response during repeated stimulation, no up-regulated key genes were detected, but 3 down-regulated genes were found suggesting that it is gene regulation that prevents the secretion of cytokines and thereby reduces the immune response. In contrast, in the case of an intolerant response, no downregulated key genes were found, but 6 up-regulated genes were identified. There is no doubt that gene regulation contributes to the increased secretion of pro-inflammatory cytokines in an intolerant response. Our data also indicate that, both in normal and intolerant responses, differences (disturbances) in gene regulation are present in the cell before the first stimulation, since after the second inflammatory stimulation, no master regulator common to all studied cytokines was found. An increase in functional reactivity during restimulation is facilitated by the increased availability of the corresponding genes for the transcriptional machinery.

It is important to find out what exactly the identified master regulators are involved in disrupting the normal immune response. It is theoretically possible to find a connection between signaling pathways as well as master regulators and the immune response. However, it appears to be more promising to conduct an experimental study on the modification of the master regulator, for example, by knockdown, followed by the study of the immune response of genetically modified cells. Such studies will reveal important diagnostic markers and new pharmacological targets for effective prevention of chronification of inflammation.

It was equally important to identify not only those genes that are directly involved in the intolerant cytokine response after inflammatory stimulation, but also the genes characteristic specifically for cells capable of exhibiting an intolerant cytokine response in the case of inflammatory stimulation. Genes that were highly expressed even before inflammatory stimulation were identified. A total of 66 such genes were identified. The top 10 of these genes will be further studied in order to establish their role in the intolerant response.

Relying only on the known functions of proteins encoded by master regulators (Supplementary Table **1**, **2**), we cannot fully explain the molecular mechanisms of intolerance of the innate immune response leading to the chronification of inflammation. This is precisely the main limitation of our work. Of course, one can put forward various speculations, but this can hardly help understanding. A much more productive way is genome editing, which leads to changes in the expression of those genes that are involved in the intolerant response. Our current work is based on this approach; however, genome editing is not an easy task, and we are using all available resources to solve it.

## CONCLUSION

We found a significant discrepancy between expression and secretion of cytokines in the intolerant response to inflammatory stimulation. It was found that normal and intolerant responses differ significantly both in terms of the number of signaling pathways involved and qualitatively since the signaling pathways for normal and intolerant responses are completely different. Master regulators characteristic of normal and intolerant inflammatory responses have been found. In the case of a normal response, no common master up-regulators were found, but on the other hand, 3 master down-regulators were identified. The reverse situation was observed with an intolerant inflammatory response. Six master up-regulators were found, but no master down-regulators were identified. An experimental study on the modification of the master regulator, for example, by knockdown, followed by the study of the immune response of genetically modified cells, should beconducted. Such studies will reveal important diagnostic markers and new pharmacological targets for effective prevention of chronification of inflammation.

## Figures and Tables

**Table 1:** Total number of signaling pathways.

**Stimulation**	**Response**	**Regulation**	**IL-1β**	**IL-6**	**CCL2**	**IL-8**
1^st^	Normal	up	41	56	66	69
		down	0	0	0	0
	Intolerant	up	68	43	68	59
		down	0	4	1	7
2^nd^	Normal	up	56	28	10	66
		down	3	0	4	1
	Intolerant	up	8	33	35	12
		down	0	0	6	1

**Note:** Up- and down-regulation was revealed by comparing transcriptomes before and after inflammatory stimulation.

**Table 2:** Master down regulators common to all cytokines in a normal inflammatory response.

**Master Molecule Name**	**Gene Symbol**	**Gene Description**
dlg3	DLG3	Discs large MAGUK scaffold protein 3
grin1	GRIN1	Glutamate ionotropic receptor NMDA type subunit 1
mmp2	MMP2	Matrix metallopeptidase 2

**Table 3:** Master up regulators common to all cytokines in an intolerant inflammatory response.

**Master Molecule Name**	**Gene Symbol**	**Gene Description**
zbtb32	ZBTB32	Zinc finger and BTB domain containing 32
osm	OSM	Oncostatin M
lbr	LBR	lamin B receptor
il3	IL3	Interleukin 3
il16	IL16	Interleukin 16
fanca	FANCA	FA complementation group A

## Data Availability

All data generated or analyzed during this study are included in this published article.

## LIST OF ABBREVIATIONS

CCL2: Chemokine (C-C motif) Ligand 2
MCP1: Monocyte Chemoattractant Protein 1
cDNA: Complementary DNA (Deoxyribonucleic Acid)
DAMPs: Damage-associated Molecular Patterns
DMSO: Dimethyl Sulfoxide
IL: Interleukin
LPS: Bacterial Lipopolysaccharide
mtDNA: Mitochondrial DNA (Deoxyribonucleic Acid)
PAMPs: Pathogen-associated Molecular Patterns
qPCR: Real-time Polymerase Chain Reaction When used Quantitatively
RNA: Ribonucleic Acid
