# Supplementary material for: Features of Gene Regulation in Violation of the Inflammatory Response of Monocyte-like Cells Bearing Mitochondrial Mutations Associated with Atherosclerosis
Source: Curr Med Chem. 2024 Sep 12;32(15):2992–3005. doi: 10.2174/0109298673303008240829075444 (PMC12376108; doi:10.2174/0109298673303008240829075444)
Supplement: Supplementary file 1 [file CMC-32-15-2992_SD1.pdf]

Supplementary Material

Features of Gene Regulation in Violation of the Inflammatory Response of Monocyte-like Cells Bearing Mitochondrial Mutations Associated with Atherosclerosis

Alexander N. Orekhov[1,*], Nikita G. Nikiforov[1], Alexander D. Zhuravlev[1], Svetlana S. Verkhova[1], Andrey V. Omelchenko[1], Daria D. Borodko[1], Vasily N. Sukhorukov[1,2], Vasily V. Sinyov[3] and Igor A. Sobenin[3]

[1]Institute of General Pathology and Pathophysiology, 8 Baltiiskaya Street, 125315, Moscow, Russia; [2]Petrovsky Russian National Center of Surgery, 2 Abrikosovsky Lane, 119991, Moscow, Russia; [3]National Medical Research Center of Cardiology, 15a Academician Chazov Street, 121552, Moscow, Russia

Table S1. Signaling pathways common for all studied cytokines.

Normal (Tolerant) Response	
**1st Stimulation**	**2nd Stimulation**
regulation of cell proliferation<br>regulation of cytokine-mediated signaling pathway<br>toll-like receptor signaling	allograft rejection<br>cellular response to lipopolysaccharide<br>chagas disease<br>graft-versus-host disease<br>inflammatory response<br>influenza a<br>negative regulation by host of viral transcription<br>nod-like receptor signaling pathway<br>positive regulation of leukocyte chemotaxis<br>positive regulation of lymphocyte migration<br>response to lipopolysaccharide<br>tnf signaling pathway<br>type i diabetes mellitus
apoptotic process<br>cellular response to molecule of bacterial origin<br>cytokineиs and inflammatory response<br>eosinophil chemotaxis<br>eosinophil migration<br>hypertrophy model<br>il1 and megakaryocytes in obesity<br>interferon type i signaling pathways<br>lipopolysaccharide-mediated signaling pathway<br>lung fibrosis<br>macrophage chemotaxis<br>macrophage migration	human cytomegalovirus infection<br>positive regulation of erk1 and erk2 cascade<br>positive regulation of hydrolase activity<br>regulation of erk1 and erk2 cascade<br>regulation of gtpase activity

monocyte chemotaxis<br>negative regulation of cytokine production<br>negative regulation of innate immune response<br>negative regulation of tyrosine phosphorylation of stat protein<br>neutrophil migration<br>perk-mediated unfolded protein response<br>positive regulation of camp-mediated signaling<br>positive regulation of i-kappab kinase/nf-kappab signaling<br>positive regulation of inflammatory response<br>positive regulation of innate immune response<br>positive regulation of interferon-alpha production<br>positive regulation of interferon-gamma biosynthetic process<br>positive regulation of interleukin-2 biosynthetic process<br>positive regulation of lymphocyte migration<br>positive regulation of nf-kappab transcription factor activity<br>positive regulation of response to biotic stimulus<br>positive regulation of rig-i signaling pathway<br>positive regulation of t cell activation<br>positive regulation of type i interferon production<br>protein adp-ribosylation<br>regulation by virus of viral protein levels in host cell<br>regulation of adiponectin secretion<br>regulation of cytokine production<br>regulation of interferon-alpha production<br>regulation of mda-5 signaling pathway<br>regulation of natural killer cell chemotaxis<br>regulation of nuclease activity<br>regulation of response to biotic stimulus<br>regulation of rna metabolic process<br>regulation of t cell proliferation<br>regulation of type i interferon production<br>response to interferon-beta<br>response to tumor necrosis factor<br>response to type i interferon<br>signal transduction through il1r<br>stat cascade<br>t cell chemotaxis<br>type iii interferon signaling	
